# Intelligent Detection of a Planetary Gearbox Composite Fault Based on Adaptive Separation and Deep Learning †

**DOI:** 10.3390/s19235222

**Published:** 2019-11-28

**Authors:** Guo-dong Sun, You-ren Wang, Can-fei Sun, Qi Jin

**Affiliations:** National Key Laboratory of Science and Technology on Helicopter Transmission, Nanjing University of Aeronautics and Astronautics, Nanjing 211106, China; sunguodong@nuaa.edu.cn (G.-d.S.); suncanfei@samri.com.cn (C.-f.S.); jq862100997@163.com (Q.J.)

**Keywords:** planetary gearbox, composite fault, adaptive separation, automatic feature extraction, intelligent detection

## Abstract

Due to the existence of multiple rotating parts in the planetary gearbox—such as the sun gear, planet gears, planet carriers, and its unique planetary motion, etc.—the vibration signals generated under multiple fault conditions are time-varying and nonstable, thus making fault diagnosis difficult. In order to solve the problem of planetary gearbox composite fault diagnosis, an improved particle swarm optimization variational mode decomposition (IPVMD) and improved convolutional neural network (I-CNN) are proposed. The method takes as input the spectrum of the original vibration signal that contains rich information. First, the automatic feature extraction of signal spectrum is performed by I-CNN, while a classifier is used to diagnose the fault modes. Second, the composite fault signal is decomposed into multiple single fault signals by adaptive variational mode, and the signal is decomposed as a model input to diagnose the single fault component. Finally, a complete intelligent diagnosis of planetary gearboxes is conducted. Through experimental verification, the composite fault diagnosis method combining IPVMD and I-CNN will diagnose the composite fault and effectively diagnose the sub-fault included in the composite fault.

## 1. Introduction

Modern machinery and equipment are developing rapidly, and tend to be large, complex and centralized. Planetary gearboxes are widely used in complex mechanical transmission systems; the occurrence of faults is random, secondary, concurrency, and concealment, and multiple fault conditions have become the norm [1,2,3]. The planetary gear and the sun gear appear as composite faults with different positions, different forms and different degrees; each fault is interfered with each other, interacted with each other, and coupled with each other. Therefore, the planetary gearbox for composite fault diagnosis is a current research difficulty. Domestic and foreign scholars have carried out a good deal of research on planetary gearbox composite faults. It is mainly processed from the perspective of fault separation, and then spectral analysis is performed based on expert experience, as described below. Liang et al. [4] proposed using a two-group sparse low-rank matrix separation method to separate the bearing and gear composite fault signals, and then solve the faults for the components. Dhamande et al. [5] proposed a composite fault diagnosis method for gears and bearings based on Continuous Wavelet Transform and Discrete Wavelet Transform. Li et al. [6] studied a differential-based employed ensemble empirical mode decomposition (EEMD) method to diagnose the composite fault of the turboexpander rotor system, establish a baseline that can successfully decouple the signal, and successfully decompose the composite fault. Yu et al. [7] performed resonance sparse decomposition on the gearbox composite fault signal to obtain different resonance components (high and low). Pan et al. [8] used the acoustic signals as source signals to diagnose gearbox composite faults. First, the adaptive generalized morphological filtering is used to denoise the signal, the principal component is extracted, then the independent component analysis is performed, and the sparse component analysis is performed on each independent component. Jia et al. [9] used to extract the composite fault characteristics of the vibration signal by the composite variational mode decomposition (VMD) and 1.5-dimensional envelope spectrum for the nonstationary operation of the rotating machine. Qin Yi et al. [10] applied the M-band flexible wavelet transform to the planetary gearbox fault diagnosis and achieved good results. Yu et al. [11] used the morphological component analysis to separate the fault characteristics of the composite signal, and then used the Hilbert envelope spectrum analysis to complete the fault diagnosis of the gearbox. Wang et al. [12] decomposed the original signal into different frequency bands and levels, and calculated the corresponding peak map by fault spectral kurtosis (SK) analysis and meshing resonance. The peak map is compared to determine the resonant frequency band caused by the fault, and the complex fault is detected by the envelope analysis method.

However, in engineering applications, a large amount of data is generated all the time, and the actual operational data in the form of composite faults contains multiple fault features. These fault features are integrated into one vector, and it is obviously impossible to complete the composite fault diagnosis by relying on expert experience. Therefore, deep neural networks with powerful automatic learning features are widely used in fault diagnosis [13,14,15,16], and have achieved fruitful results. Yong Li et al. [17] proposed a planetary gear fault diagnosis method based on variational mode decomposition and deep neural network (DNN) power spectral entropy. Qin Yi et al. [18] proposed an improved deep confidence network in the fault diagnosis of wind turbine planetary gearbox, and achieved good results. Lei et al. [19] performed sparse filtering on deep neural networks to achieve automatic feature extraction of bearing signals and complete fault identification. Hung Nguyen et al. [20] performed EEMD decomposition on the original vibration signal, statistically analyzed the time domain and frequency domain parameters of the component signal, and used DLN to realize the self-learning of the feature parameters, and finally used the classifier to complete the diagnosis. Zhang, X. et al. [21] performed narrow-band interference cancellation processing on the original signal, and then extracted multifractal features in the signal through multi-fractal, and uses whale optimization algorithm (WOASVM) to complete the composite fault pattern recognition. Xiaolian Liu et al. [22] used Deep Forest to perform automatic feature extraction on the original experimental signal with noise added, and cascaded different types of fault features for learning and classification, which is robust to noise and is not limited by the amount of training data. Xin Wang et al. [23] proposed the application of SAE and DBN in the fault diagnosis of rotating machinery, and applied a new activation function to obtain better results. Shao et al. [24] used the compressed sensing technology to reduce the dimensionality of the vibration signal, and the Gaussian visible unit and the exponential moving average technique improved the convolution depth confidence network, and finally realized the feature extraction of the rolling bearing signal. Zhang et al. [25] integrated Dropout and batch normalization operations in the training process of deep convolutional neural networks to improve the antinoise performance and convergence speed of the network, and achieve adaptive extraction of bearing fault features.

Through the above analysis, in the case of multiple component failures occurring in the planetary gearbox, the weaker fault features are often submerged in the stronger fault features. The existing intelligent composite fault diagnosis methods mostly regard it as a mode, and demodulation analysis of vibration signals to diagnose faults often leads to missed or misjudgment and relies too much on expert experience. Therefore, to complete the intelligent diagnosis of the planetary gearbox composite fault, two points must be achieved: (1) complete the adaptive separation of the composite fault signal and the decomposition of the composite fault signal to the single fault signal; (2) realize the effective automatic feature learning of the composite fault signal, thus evading expert experience.

In this paper, a diagnostic method based on adaptive fault separation combined with I-CNN is proposed for composite faults. Firstly, a single fault or compound fault is classified and identified. Secondly, adaptive variational mode decomposition is used to decompose the complex fault signal into multiple single fault signals, and the decomposed signal is used as the single fault component of the model input diagnosis. Finally, the complex fault intelligent diagnosis of planetary gearbox is completed. The method is applied to the analysis of experimental vibration signals. The experimental results show that the method overcomes the dependence on signal preprocessing and artificial feature extraction. It is more effective and reliable than the traditional method, and truly completes the intelligent diagnosis of complex faults. According to the literature research, the method proposed in this paper is the first attempt on the planetary gearbox composite fault diagnosis. The main research of this paper is summarized as follows.

(1) In order to prevent over-fitting of CNN, in the training phase, the input feature vector is pooled using the maximum pooled Dropout method; in the model, the average is combined with the retention probability and the unit value probability weight to obtain a better recognition rate.

(2) For the uncertainty of parameters in VMD decomposition, it is proposed to use the improved PSO method to optimize the number of parameter modal components K and the second penalty factor α, and automatically select the best combination of parameters.

(3) The effective combination of IPVMD adaptive separation and I-CNN provides a new method for intelligent diagnosis of planetary gearbox composite faults.

The rest of the article is as follows: Section 2 describes the theory of I-CNN and IPVMD. The effectiveness of the composite fault data verification method is used in Section 3. Finally, the conclusion is provided in Section 4.

## 2. Method Theory

### 2.1. Improved PSO Optimized VMD

The variational mode decomposition theory shows that the method for decomposing VMD requires a preset number of parameters, a K modal component of the secondary penalty factor α, and produces results with a greater impact [26]. How to choose the appropriate two input parameters is the premise and key for VMD to accurately decompose the signal. As a swarm intelligence optimization algorithm, particle swarm optimization has good global optimization ability. Therefore, this paper optimizes the parameters by using improved PSO. In the optimization process, the particle update strategy and inertia weight are improved, and the optimal parameter combination is automatically selected.

An adaptive function needs to be determined during the particle update process. Entropy can evaluate the sparse characteristics of the signal effectively; the size of the entropy reflects the uncertainty of the signal. The greater the entropy value, the greater uncertainty of the signal.

**Figure 1:**
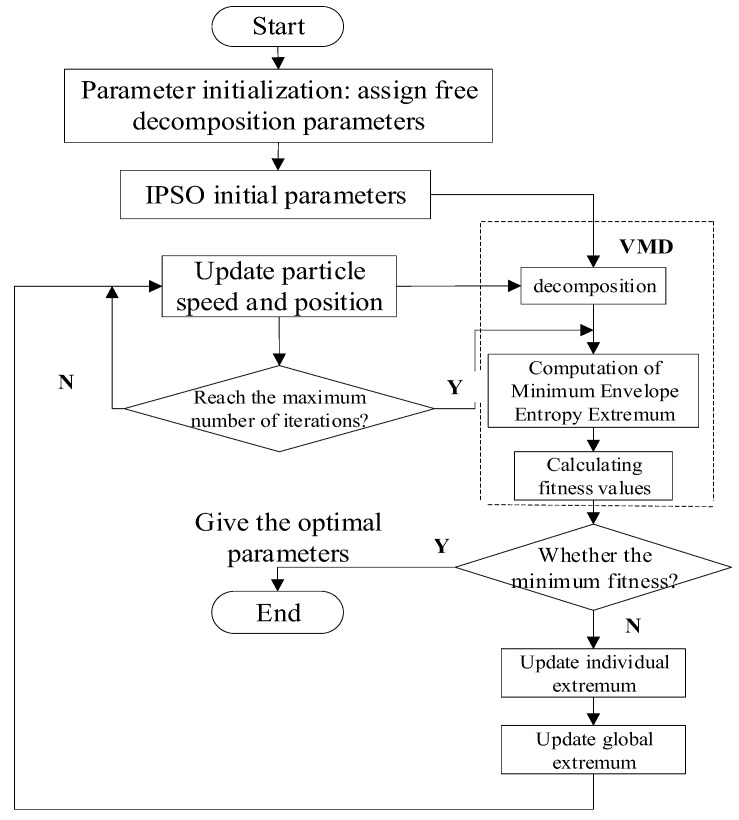
Adaptive variational mode decomposition process.

The demodulated and decomposed envelope signal sequence *e_j_*, the entropy value of which is the envelope entropy, can reflect the sparse characteristics of the original signal. Therefore, the envelope entropy *E_e_* is used as an adaptive function. The specific expression is as follows:

(1) $$\left\{ \begin{array}{l} E_{e}=-\sum_{j=1}^{N}e_{j}\mathrm{lg}e_{j}\\ e_{j}=a(j)/\sum_{j=1}^{N}a(j) \end{array}\right.$$

where *e_j_* is the normalized form of *a*(*j*), and *a*(*j*) is the envelope spectrum signal of signal *x*(*j*). The specific optimization process of VMD is shown in Figure 1.

### 2.2. Improved Convolutional Neural Network Theory

**Figure 2:**
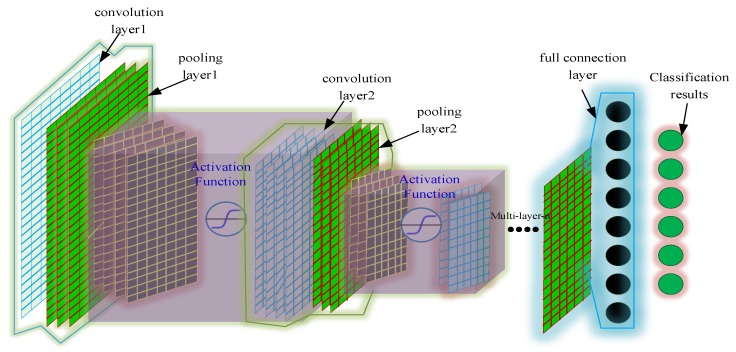
Schematic diagram of convolutional neural network.

Convolutional neural networks [27] are composed of many neural network layers. The two different types of layers—convolution and pooling—are usually alternated. The depth of each filter in the network is added from left to right, and finally consists of one or more fully connected layers, as shown in Figure 2. Each convolutional layer in a convolutional neural network consists of several convolutional units, which the parameters of each convolutional unit are optimized by a backpropagation algorithm. The purpose of the convolution operation is to extract different features from the input. The first convolutional layer may only extract some low-level features such as edges, lines, and corners, and more convolutional layers can iteratively extract more complex features from lower-level features.

#### Pooling Layer Improvement

Usually, the feature dimension obtained after the convolution layer is very large, and the feature needs to be cut into several regions, and the maximum value or average value is taken to obtain a new feature with a small dimension. The purpose is to reduce the feature map. The main function is to reduce the amount of calculation by reducing the parameters of the network, and to control the over-fitting to a certain extent; that is, a pooling layer is added after the convolution layer is needed. An ideal way of pooling can effectively remove irrelevant information; commonly used processing methods are pooled and the average maximum pooled. Pooling may dilute the average effect of a high unit value. Pooling the maximum value is only taken of the maximum value of each pooled area, ignoring the role of other cell values in the region. The above two drawbacks can be avoided using Dropout. Therefore, the following improvements have been made:

(1) Maximum pooling Dropout in the training phase.

Dropout is used in the pooling layer during the training phase, the forward propagation expression is as follows:

(2) $$\overset{\wedge }{a_{j}^{n}}∼m_{j}^{n}⊗a_{j}^{n}$$

(3) $$a_{j}^{n+1}=pool(\overset{\wedge }{a_{j,1}^{n}},\cdots ,\overset{\wedge }{a_{j,i}^{n}},\cdots ,\overset{\wedge }{a_{j,l}^{n}})$$

where “⊗” is the element corresponding multiplication, *n* is the number of neurons, *j* is the *j*-th pooling unit, *m* is a binary mask, obeys the Bernoulli distribution, and the mask is multiplied by the input $a_{j}^{n}$ of the layer to obtain the modified eigenvector $\overset{\wedge }{a_{j}^{n}}$, and then the vector $\overset{\wedge }{a_{j}^{n}}$ is pooled, using the maximum pooled dropout method for pooling operations.

(2) Probability-weighted model average in the test phase.

The maximum pooled Dropout method is used in the training phase. The test phase [28] suggests that if the activation value $a_{j,i}^{n}$ is retained, the theoretical probability can be expressed as:

(4) $$Pr(a_{j}^{n+1}=a_{j,i}^{n})=p_{j,i}=pq^{l-1}$$

where *p* is the retention probability and *q* = 1 − *p* is the probability of suppression.

In the test phase, the model average is used, and the value of each pooled region is obtained by Equation (5).

(5) $$a_{j}^{n+1}=\sum_{i=0}^{l}p_{j,i}a_{j,i}^{n}=\sum_{i=1}^{l}p_{j,i}a_{j,i}^{n}$$

where *i* = 0 is the pool area is dropout, which is probabilistic weighted pooling.

In the training phase of this paper, the input feature matrix is modified with a binary mask obeying the multipattern distribution. In the test phase, the influence of the probability of each unit value in the pool region is considered, which overcomes the problem of over-fitting. In the training phase, assuming the *j*-th pooled area of the nth layer, the maximum pooled Dropout method is used.

(6) $$a_{j}^{n+1}=\max(\overset{\wedge }{a_{j,1}^{n}},\cdots ,\overset{\wedge }{a_{j,i}^{n}},\cdots ,\overset{\wedge }{a_{j,l}^{n}})$$

In the test phase, the probability *p_j,i_* of the value of each neuron in the input feature vector over the entire pooled area, expressed as follows:

(7) $$p_{j,i}=\frac{a_{j,i}^{n}}{\sum_{k=1}^{l}a_{j,k}^{n}}$$

In this paper, taking into account the impact probability *p_j,i_* and retention probability *p*, proposed a new method of model averaging. First, the pooled area is multiplied by *p* and multiplied by its probability *p_j,i_*. Then, the summation of all the values in the pooled area is the value obtained after the pooling operation.

(8) $$a_{j}^{n+1}=\sum_{i=1}^{l}pp_{j,i}a_{j,i}^{n}$$

Taking this value as a pooled area, an approximation of the average of all possible models is considered.

### 2.3. Intelligent Diagnosis Procedure of This Article

**Figure 3:**
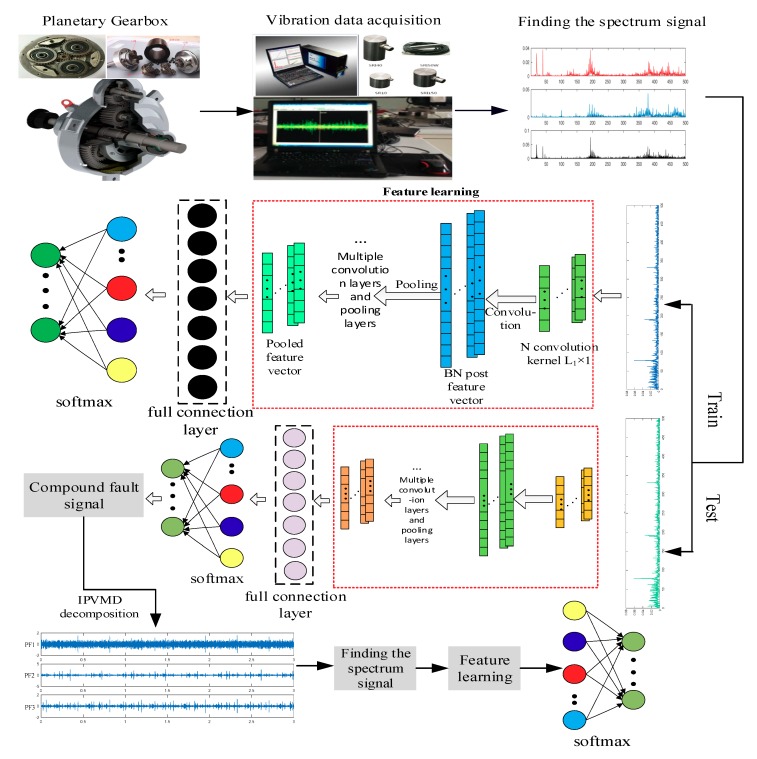
Flowchart of composite fault diagnosis method combining IPVMD and I-CNN.

This paper proposes a composite fault diagnosis method that combines IPVMD and I-CNN. The flow chart of the method is shown in Figure 3. The main steps are as follows:

Step 1: The planetary gear train vibration signal is measured by the sensor and collected by the data acquisition system.

Step 2: Perform spectrum and envelope spectrum analysis on the collected vibration data, determine the validity of the data, and obtain training samples and test samples of the data set composed of the spectrum vibration data.

Step 3: Determine the number of iterations of the I-CNN model, the training batch and the parameters of each layer, and complete the model training; then, input the learned test sample features into the Softmax classifier for the planetary gear train failure mode identification.

Step 4: Determine the composite failure mode by step 3, adaptively decompose it by IPVMD, then obtain the single fault component to form Data Set 2, and analyze the decomposition effectiveness.

Step 5: Input Data Set 2 directly into the I-CNN model for automatic feature extraction, and input the learned features into the Softmax classifier to complete the composite fault diagnosis of the planetary gear train.

## 3. Results

### 3.1. Test Conditions and Fault Settings

**Figure 4:**
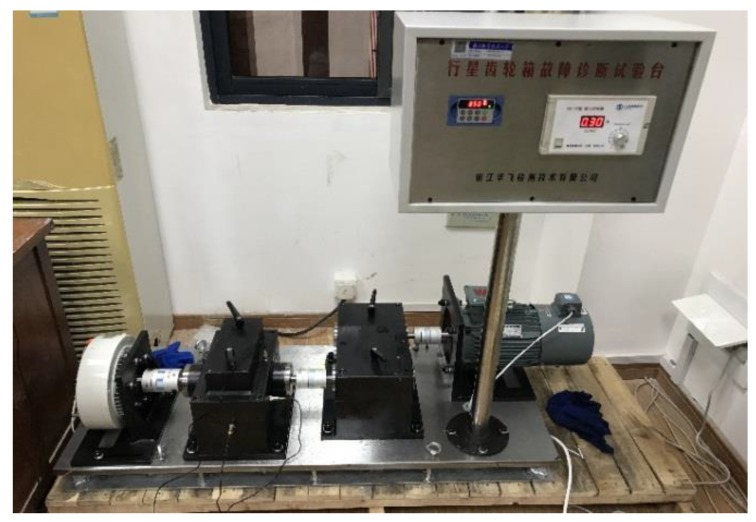
Planetary gearbox experiment platform.

Effectiveness and advantages of the composite planetary gearbox fault diagnosis method proposed is described, using experimental data collected in the laboratory planetary gearbox. The test-stage planetary gearbox is driven by a three-phase motor, a programmable controller, II helical gear box, planetary gearbox stage I and magnetic loader or the like, as shown in Figure 4. The parameters of each gear box of the experimental platform are shown in Table 1.

**Figure 5:**
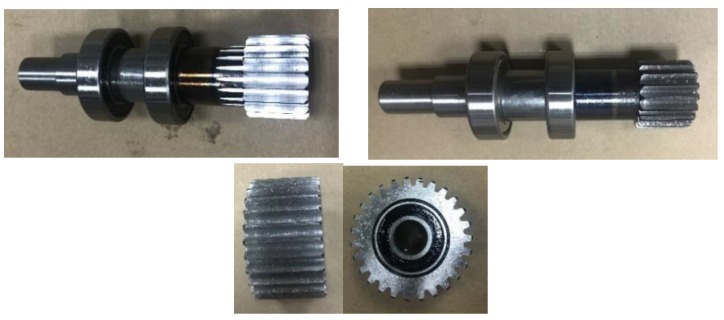
Simulated fault mode components.

Experiments provided, two kinds of sun gear fault modes: sun gear wear(SW) and sun gear cracks(SC); two kinds of planetary gear failure modes: planetary gear wear(PW) and planetary gear cracks(PC); two types of composite failure modes: PC-SW, PW-PC-SC, as shown in Figure 5. The planetary gearbox vibration signal of 209 working conditions was collected by the acceleration sensor, 19 kinds of rotation speed (600 rpm–1500 rpm), and 11 kinds of load (0 N. m–30 N. m). The sampling frequency is 20,480 Hz, the number of data samples collected in each state mode is 1672, and 8 data samples are collected for each working condition. That is, 11,704 planetary gearbox samples are collected under the normal state of the planetary gearbox and the four failure modes data.

### 3.2. Data Description

This paper sets a total of two data sets. Data Set 1: In each state mode, 1322 data samples are randomly selected to form a training data set, and the remaining 350 data samples constitute a test data set. The test data set sample size is 2450, and the training data set sample size is 9254, as shown in Table 2. 

Data Set 2: The result judged by Data Set 1 through the method mentioned above. IPVMD decomposition is used to decompose the complex fault signal to obtain the single fault PF (product function) component signal. A labeled Data Set 2 is constructed, as shown in Table 3.

### 3.3. Experimental Results and Analysis

#### 3.3.1. Data Set 1 Experiment

**Figure 6:**
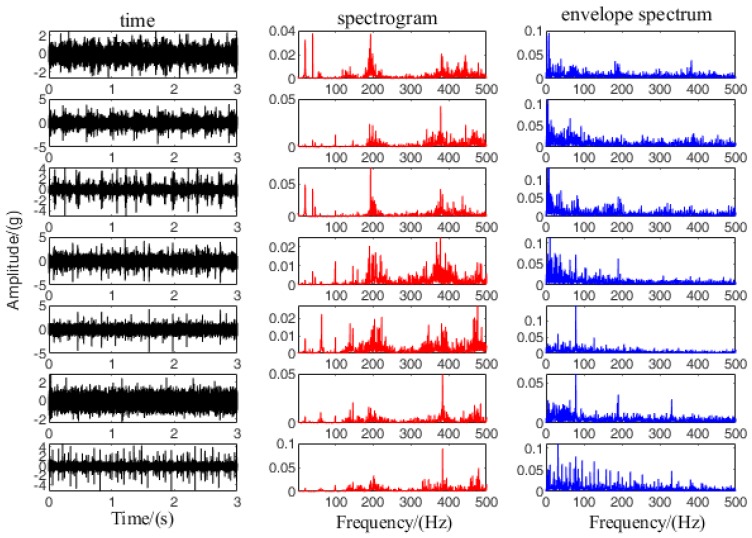
Map of vibration signal of Data Set 1.

This section uses the above Data Set 1 to perform performance testing on I-CNN. In order to effectively realize the vibration signal for feature automatic extraction, the spectrum and the envelope spectrum are obtained from the original vibration signal. The vibration signal at 1200 rpm and 0 N. m load as shown in Figure 6, the time domain data of 3 s is intercepted, and the spectrogram and envelope spectrum are shown as the first 500 Hz. Firstly, the first 2000 data points of the frequency domain are used as input to the I-CNN. Secondly, the CNN hierarchy is selected as input-C1-S1-C2-S2-C3-S3-FC. Finally, the parameter values of each layer are determined, and the detailed parameter values of CNN are shown in Table 4.

**Figure 7:**
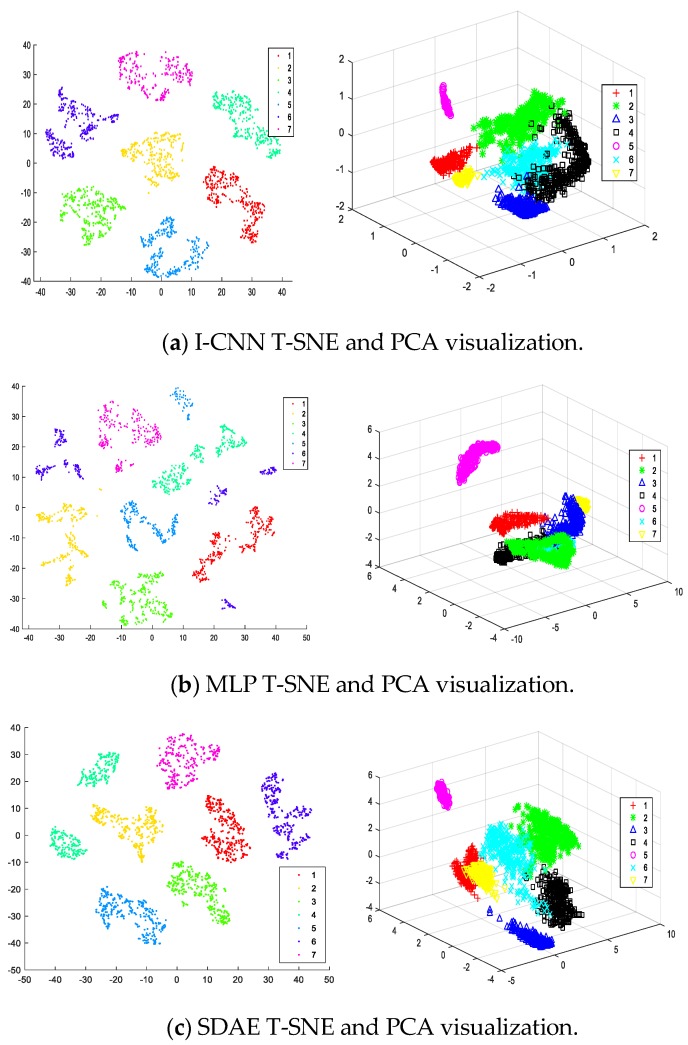
T-SNE and PCA visualization of I-CNN, MLP, SDAE.

Analysis of the feature validity of I-CNN network extraction through experiments, the features extracted by the I-CNN network are analyzed by principal component analysis (PCA) [29] and t-distributed stochastic neighbor embedding (T-SNE) [30]. For the superiority of the comparison method, selecting multi-layer perceptron (MLP) [31] and spare denoising autoencoder (SDAE) [32] for comparative analysis for the superiority of the comparison method. The visualization results are shown in Figure 7.

Figure 7a is a feature visualization scatter plot automatically extracted by the I-CNN network. It is apparent in the figure that each state pattern feature of the planetary gearbox is highly concentrated. The distance between different state modes is obvious, and the aggregation mode has regularity and regionality.

MLP and SDAE are compared as experimental results, the T-SNE and PCA visual scatter plots of the features were automatically extracted as shown in Figure 7b,c. It can be seen from Figure 7b that although the different state modes of the gears are effectively distinguished in the area, the degree of scatter is not high, and there is a small part of the aliasing, especially in state 6 (planetary crack-sun gear wear). In the results of Figure 7c, the characteristics of SDAE extraction are significantly better than the MLP method, and the scatter points of each state mode exhibit an aggregation mode. Compared with the I-CNN method, each state mode is not very concentrated, and there are partial intersections and overlaps between different state modes.

The above analysis results show that the I-CNN method is very effective for extracting the features of Data Set 1. It is easier to distinguish between the T-SNE and PCA visualization maps than the MLP method and the SDAE method, which is helpful for fault diagnosis based on features.

**Figure 8:**
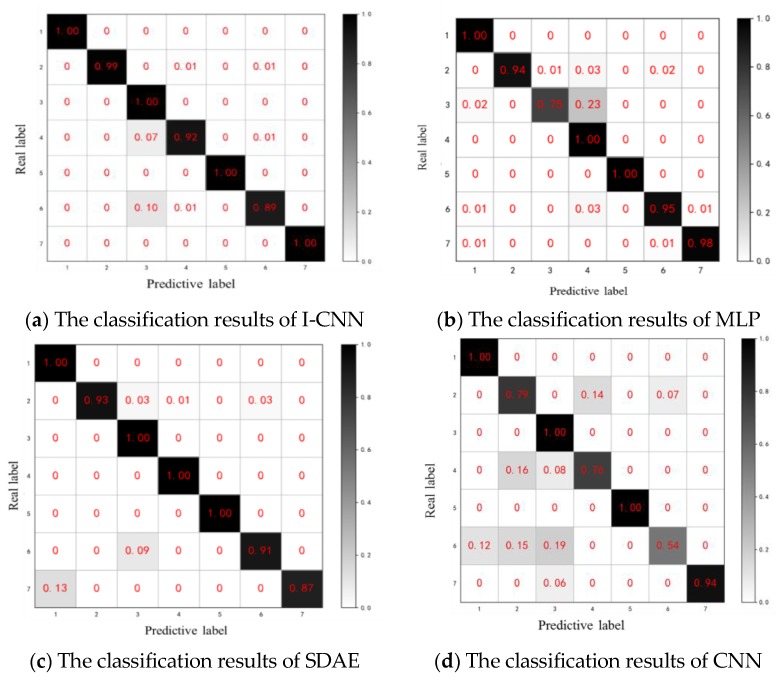
Classification results of three fault diagnosis methods.

The classification results of the three methods of I-CNN, MLP and SDAE are shown in Figure 8.

In the results of Figure 8a that the features extracted by the I-CNN method can be well classified by Softmax with an accuracy of 97.1%, in which the sun wheel wear mode and the composite fault 1 are poorly classified, and most of them are misclassified into cracks. Since cracks and wear are minor early faults, the fault characteristics are similar and confusing. Because the characteristic frequency of the wear fault is the distributed fault characteristic frequency, and the composite fault 1 contains the sun gear wear fault, it is greatly affected, and the confusion is not easy to distinguish.

As can be seen from Figure 8b–d, the results of the MLP method and the SDAE method are similar to those of the I-CNN method described above. Among them, the MLP method mainly focuses on the sun wheel wear mode, which is misclassified into the planetary wheel crack mode with an accuracy of 94.6%. The SDAE method mainly focuses on the composite failure mode with an accuracy of 95.8%. The CNN method mainly focuses on the composite failure mode with an accuracy of 88%. A comparison table of diagnostic accuracy and convergence speed between the four deep neural networks is shown in Table 5. Therefore, the feature diagnosis results extracted by MLP and SDAE methods are not as good as the I-CNN method, and there are many misclassification results, which bring a good deal of uncertainty to the subsequent diagnosis.

**Figure 9:**
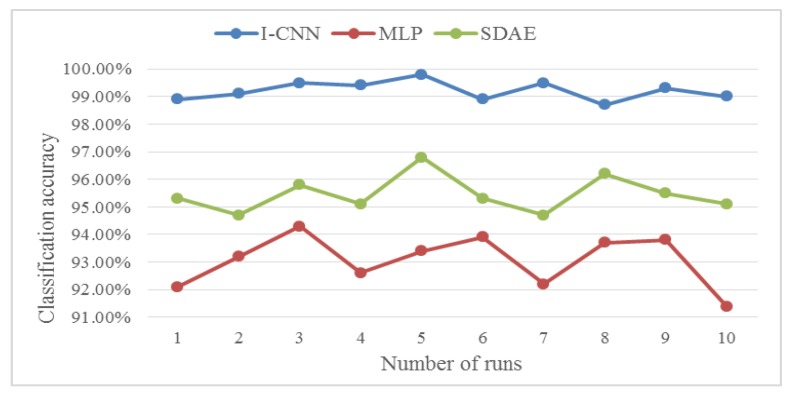
Classification accuracy results for ten trials.

Considering the randomness and instability of the experiment, 10 experimental results were processed, and the obtained classification accuracy is shown in Figure 9. The I-CNN automatic feature extraction method proposed in this paper is obtained from the polyline in the figure. The classification accuracy and stability are higher than MLP and SDAE.

#### 3.3.2. Data Set 2 Experiment

**Figure 10:**
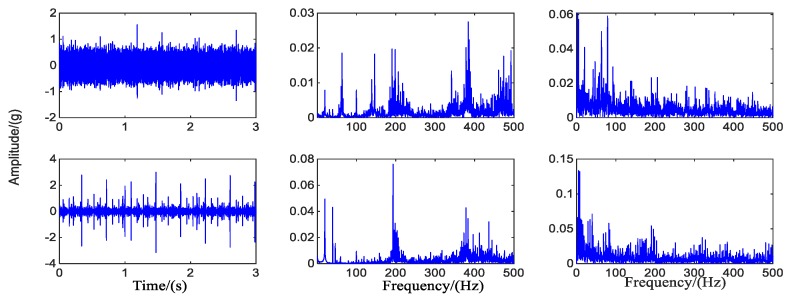
State mode 6 decomposition results, spectrum and envelope spectrum.

**Figure 11:**
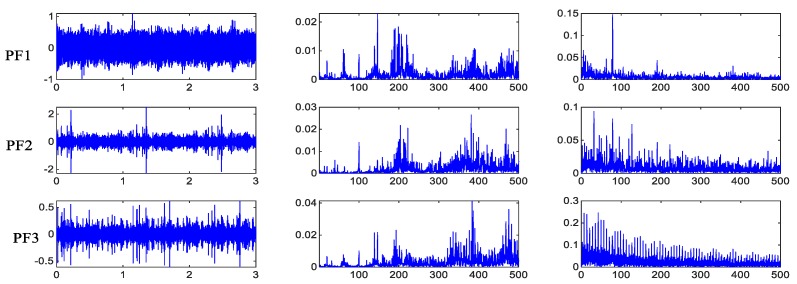
State mode 7 decomposition results, spectrum and envelope spectrum.

**Figure 12:**
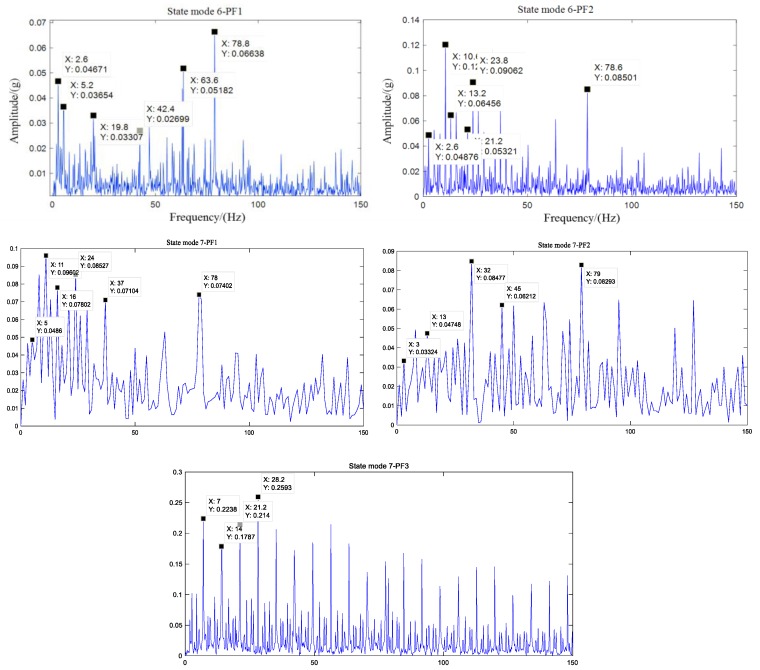
Envelope spectrum results of each PF component.

The experimental results of Data Set 1 can diagnose that the state mode of 6 and 7 is a composite fault, but it cannot be diagnosed that several fault modes are included. Therefore, the IPVMD method proposed in this paper is adaptively decomposed to obtain the corresponding PF component, and the spectrum and envelope spectrum are obtained for the corresponding PF components. The rated speed of 1200 rpm and 27 N. m are a result of the load shown in Figure 10 and Figure 11, the time domain data of 3 s is intercepted, and the spectrogram and envelope spectrum are shown as the first 500 Hz. To initially verify the decomposition effect and success rate of the IPVMD, the envelope spectrum is amplified to analyze the fault frequency of the PF component. The result is shown in Figure 12.

In the envelope spectrum results of Figure 12, The spectral lines of the respective frequency and fault frequencies can be clearly seen. Due to the modulation effect of the planet carrier, its multiple frequency (2.6 Hz), double frequency, etc., appear in the envelope spectrum, and the fault frequency exhibits the spectral line of adding and subtracting the frequency of the planet carrier. Since the composite fault from the planetary gear train vibration signal is usually complicated in reality, it is excited by multiple vibration sources, so there is a slight deviation between the obtained frequency and the theoretical frequency. Through the above analysis, it can be concluded that the decomposition result of IPVMD is effective, and it can be used as an input to improve CNN to perform composite fault diagnosis.

**Figure 13:**
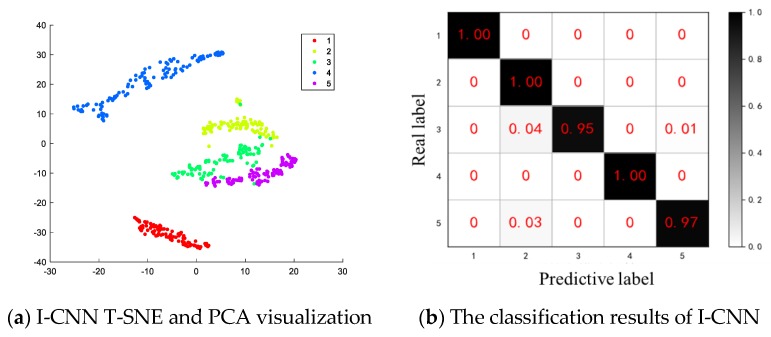
Visualization and classification results of I-CNN.

In this paper, the composite fault data in the Data Set 1 test set, the IPVMD decomposition result constitutes the Data Set 2, and the spectrum of each component is obtained as the input of the I-CNN network, and it is directly tested without training; the results shown in Figure 13. In the results of Figure 13a, the same data state features of the I-CNN network for extracting the planetary gearbox are well clustered, the different state modes are effectively separated, and a regular aggregation mode is presented. It can be seen from Figure 13b that the features extracted by the improved CNN method can be better classified by Softmax with an accuracy of 98.6%. According to the decomposition results, 350 of the normal mode, 350 of the sun gear crack mode, and 700 of the planetary gear crack modes are successfully identified; a few of the sun gear wear mode and the planetary gear wear mode are misclassified into the sun gear crack mode.

**Figure 14:**
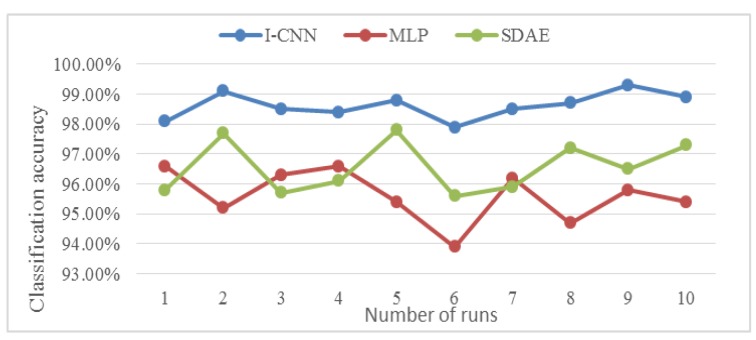
Classification accuracy results for ten trials.

Considering the randomness and instability of the experiment, 10 experimental results were processed, and the obtained classification accuracy is shown in Figure 14. The I-CNN automatic feature extraction method proposed in this paper is obtained from the polyline in the figure. The classification accuracy and stability are higher than MLP and SDAE.

## 4. Conclusions

For the planetary gear trains in the planetary gearbox, the weak fault features are often submerged in the strong fault characteristics. Direct demodulation analysis of vibration signals to diagnose faults usually leads to misdetection or misjudgment, and relies too much on expert experience. Most of the existing intelligent composite fault diagnosis methods regard it as a mode. In this paper, an adaptive fault separation IPVMD combined with I-CNN for planetary gearbox composite fault diagnosis is proposed. The main conclusions can be drawn as follows:

(1) IPVMD can adaptively decompose the vibration signal of the planetary gearbox, complete the decomposition of the composite fault signal to the single fault signal, reduce the dependence on the expert experience, and provide guarantee for the subsequent feature extraction.

(2) Compared with the commonly used deep neural network, the features extracted by I-CNN are obviously effective as show in the PCA and T-SNE diagrams, the feature scatter aggregation in the same state mode is very high, the separation distance is obvious in different state modes, and the discrimination of different failure modes is good.

(3) Experimental results show that the method overcomes the dependence on signal preprocessing and artificial feature extraction. The proposed method is more efficient and reliable than traditional methods, and can truly complete the intelligent diagnosis of composite faults.

## Figures and Tables

**Table 1:** Experimental platform gearbox parameters.

Gearbox	Parameter
Helical gearbox first stage	Small gear teeth 40, large gear teeth 50
Helical gearbox second stage	Small gear teeth 50, large gear teeth 60
Planetary gearbox	Ring gear teeth 72; Planetary wheel teeth 27; Sun gear teeth 18

**Table 2:** Data Set 1.

Serial Number	State Mode	Number of Data	Number of Training	Number of Testing	Operating Conditions
Mode 1	normal status	1672	1322	350	209
Mode 2	SC status	1672	1322	350	209
Mode 3	SW status	1672	1322	350	209
Mode 4	PC status	1672	1322	350	209
Mode 5	PW status	1672	1322	350	209
Mode 6	PC-SW status	1672	1322	350	209
Mode 7	PW-PC-SC status	1672	1322	350	209

**Table 3:** Data Set 2.

State Mode	Samples	Testing Set	Operating Conditions
normal status	350	350	209
Mode 6_PF1	350	350	209
Mode 6_PF2	350	350	209
Mode 7_PF1	350	350	209
Mode 7_PF2	350	350	209
Mode 7_PF3	350	350	209

**Table 4:** Description of the model parameters.

I-CNN Layer	Parameters
Convolution 1	Kernel size: 5; Number of convolution kernels 12; Step size: 2
Pooling 1	Pooling area: 4; Step size: 2
Convolution2	Kernel size: 5; Number of convolution kernels: 6; Step size: 2
Pooling2	Pooling area: 2; Step size: 2
Convolution3	Kernel size: 3; Number of convolution kernels: 12; Step size: 2
Pooling3	Pooling area: 2; Step size: 2
Other parameters	Maximum iterations: 100; learning rate: 1; Batch size: 128

**Table 5:** Comparison of diagnostic accuracy and convergence speed.

Method	I-CNN	CNN	MLP	SDAE
Diagnostic accuracy (%)	97.1	88	94.6	95.8
Time (seconds/s)	39.49	41.41	38.36	123.74
